# The Effects of Protein and Carbohydrate Supplementation, with and without Creatine, on Occupational Performance in Firefighters

**DOI:** 10.3390/nu15245134

**Published:** 2023-12-18

**Authors:** Kaia Elstad, Conley Malone, Joel Luedke, Salvador J. Jaime, Ward C. Dobbs, Thomas Almonroeder, Chad M. Kerksick, Adam Markert, Andrew R. Jagim

**Affiliations:** 1 Exercise & Sport Science Department, University of Wisconsin-La Crosse, La Crosse, WI 54601, USAwdobbs@uwlax.edu (W.C.D.); 2 Medicine & Health Sciences, Des Moines University, Des Moines, IA 50312, USA; 3 Sports Medicine Department, Mayo Clinic Health System, La Crosse, WI 54650, USAckerksick@lindenwood.edu (C.M.K.); 4 Department of Physical Therapy, Trine University, Angola, IN 46703, USA; almonroedert@trine.edu; 5 Exercise and Performance Nutrition Laboratory, Department of Kinesiology, Lindenwood University, St. Charles, MO 63301, USA; 6 La Crosse Fire Department, City of La Crosse, La Crosse, WI 54601, USA; markerta@cityoflacrosse.org

**Keywords:** tactical athletes, ergogenic aids, firefighter performance

## Abstract

Background: The purpose of this study was to assess the effects of protein and carbohydrate supplementation, with and without creatine, on occupational performance in firefighters. Methods: Using a randomized, double-blind approach, thirty male firefighters (age: 34.4 ± 8.4 yrs., height: 1.82 ± 0.07 m; weight: 88.6 ± 12.5 kg; BF%: 17.2 ± 5.8%) were randomized to receive either (A.) 25 g of whey protein isolate + 25 g of carbohydrate powder (ProCarb group); or (B.) ProCarb + 5 g of creatine (Creatine group) in a double-blind fashion over a period of 21–26 days (depending on shift rotations) to evaluate the impact of supplementation on occupation-specific performance. At baseline and following supplementation, firefighters completed a battery of tests. These tests included an aerobic speed test on an air-braked cycle ergometer followed by the hose carry, body drag, stair climb, and Keiser sled hammer for time. Results: No significant differences in measures of performance were observed at baseline (*p* > 0.05). There was a significant main effect for time observed for rescue, stair climb, total time to completion, and time trial performance (*p* < 0.05). There was a significant group × time (*p* < 0.05) interaction for rescue and forcible entry. Independent sample *t*-tests indicated that the Creatine group experienced a greater reduction (from baseline) in completion time for the rescue (1.78 ± 0.57 s, 95% CI: 0.61, 2.95 s, *p* = 0.004) and forcible entry (2.66 ± 0.97 s, 95% CI: 0.68, 4.65 s, *p* = 0.01) tests compared to the ProCarb group. No significant group × time interactions were observed for the hose line advance, stair climb, total time to completion, and time trial performance (*p* > 0.05). Conclusions: The addition of supplemental creatine to a protein and carbohydrate supplement to the diet of career firefighters throughout a three week period improves occupational performance in firefighters in specific areas of high-intensity, repetitive actions.

## 1. Introduction

Firefighters are viewed as tactical athletes due to the physically demanding nature of the occupation and the specialized activities they routinely complete. Firefighters repeatedly perform high-intensity functional tasks at varying intervals, and are often exposed to high-temperature environments and environmental hazards, which places a high degree of physiological and thermoregulatory strain on the body [1,2]. Previous research has utilized occupation-specific assessments and fire suppression simulations as a way to help characterize the physical demands of firefighting tasks, which are routinely performed at intensities that correspond to 70–73% of maximal aerobic capacity and ~90% of maximal heart rate [3,4]. Various physical factors have also been shown to be associated with measures of occupational performance. For instance, physical characteristics such as abdominal strength, upper body muscular strength and endurance, and anaerobic power have been associated with higher ratings of performance during occupational performance tests [5,6,7]. As such, there is a need for specialized physical training, fitness requirements, and nutritional strategies.

A recent position stand outlined the importance of adequate energy and macronutrient intakes to meet the activity demands of tactical athletes [8]. Further, previous research has also examined the potential performance benefits following ingestion of dietary supplements in tactical populations [9,10,11,12,13]. It is recommended that firefighters follow dietary guidelines established for active individuals, which include a higher protein intake (1.4–1.6 g·kg·day^−1^) than the current recommended daily allowance for the general population (0.8 g·kg·day^−1^) to help support the need for recovery and maintenance of lean body mass [8]. Additionally, previous work in non-tactical populations has found the addition of carbohydrates to protein may augment amino acid uptake, attenuate post-exercise muscle damage, and aid in recovery, while also further enhancing glycogen availability during subsequent activity [14,15,16,17]. For example, the addition of carbohydrates (~35 g) to amino acids (~6 g of a balanced amino acid mixture) has been shown to produce a net muscle protein synthetic response that was roughly equivalent to the sum of the independent effect of either nutrient in isolation [18], suggesting there may be synergistic benefits of combining protein and carbohydrates together. These benefits appear to extend to master’s athletes (≥35 years) as well as the consumption of carbohydrates and carbohydrates + protein after intense endurance exercise elevated performance to a greater degree than supplementing with water + electrolytes [19]. However, the specific influence of such nutritional interventions on occupational performance in firefighters is currently unknown.

Creatine may be another nutrient of interest for tactical populations because of its well-supported ergogenic benefits and minimal reported side effects across the literature [13,20,21]. Creatine supplementation has been shown to increase intramuscular phosphocreatine stores [21,22], which in turn can increase the ability to synthesize (and re-phosphorylate adenosine diphosphate) adenosine triphosphate (ATP) [21]; thereby, playing a vital role in cellular metabolism during periods of intermittent, high-intensity exercise [20,23,24]. In addition, creatine supplementation has been shown to enhance recovery time in between bouts of activity [25] and has also been proposed to aid in thermoregulation during physical activity in hot and humid environmental conditions [26], which may confer additional benefits to firefighters during fire suppression activities. While strong evidence is available in support of the ergogenic benefits of creatine for a variety of active populations and across a wide array of physical performance parameters, less information is available regarding the benefits of creatine supplementation for occupational performance in firefighters. Specifically, mixed results have been reported regarding the ergogenic benefits of creatine in tactical populations [9,12,13,27]. While some studies have reported improvements in select measures of performance (including performance of occupation-specific tasks) [9,12], others have reported null findings [13,27]. Because of the anaerobic nature of firefighter-specific tasks [5,6,28,29], it is plausible that creatine supplementation could confer ergogenic benefits both when used in isolation and when added to a fitness program. For example, tasks commonly used during firefighter trainings and simulations have been shown to elicit a heart rate response close to the maximum heart rate, require a high degree of muscular strength and endurance, and are often completed in less than 30 s. All of these descriptions help to provide a rationale as to the potential role that creatine, specifically intramuscular phosphocreatine stores, may have in augmenting short-duration performance during these firefighting tasks.

Previous work has examined the effects of adding creatine to whey protein and carbohydrates on training adaptations in resistance-trained individuals with mixed findings [30,31,32,33], yet little information is available regarding the performance benefits of this supplementation strategy in firefighters. There is a continued need to explore performance nutrition strategies pertaining to not only supporting physical activities of firefighting but also those tailored toward master’s athletes as the average age of career firefighters tends to be in the mid-40s [34]. While previous research tends to report favorable improvements in performance, recovery, and training adaptations following protein and carbohydrate supplementation, with or without creatine, the ergogenic and occupation-specific benefit of these ingredients in firefighters is largely unknown. Therefore, the primary aim of the current study was to examine the effects of protein and carbohydrate supplementation, with or without creatine, on occupational performance in firefighters. We hypothesized that the addition of creatine would promote greater improvements in firefighter-specific occupational performance.

## 2. Materials and Methods

### 2.1. Study Design

**Figure 1:**
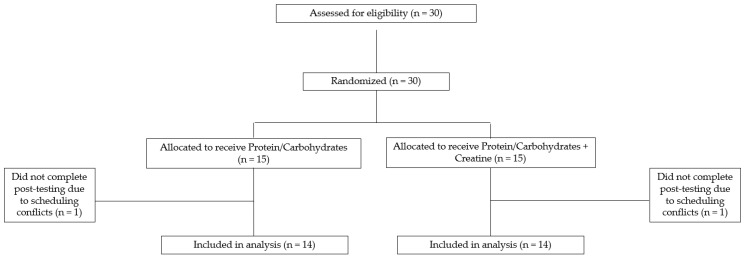
CONSORT diagram.

In a randomized, parallel-group, double-blind fashion, active-duty firefighters were randomly assigned to ingest (A) whey protein isolate + carbohydrate (ProCarb Group); or (B) whey protein isolate + carbohydrate + creatine monohydrate (Creatine Group) for a 21–26-day supplementation period (the ClinicalTrials.gov Identifier is: NCT06172543). A CONSORT diagram is presented in Figure 1. At baseline, firefighters completed a battery of occupation-specific tests at a training site in the morning while on shift. On the opposite day of their 48 h shift, firefighters completed a 3.5 km time trial at their designated fire station. Participants were asked to resume their regular dietary habits and weekly activity routine throughout the study period. Following the supplementation period, firefighters repeated all baseline testing procedures. Due to rotation shifts, post-testing was scheduled during a shift that best aligned with 24 days post-baseline testing (average supplementation duration was 23 ± 2 days; minimum days = 21, maximum days = 26).

### 2.2. Participants

Thirty active-duty male structural firefighters were enrolled (age: 34.4 ± 8.4 yrs., height: 1.82 ± 0.07 m; weight: 88.6 ± 12.5 kg; BF%: 17.2 ± 5.8%) in the current study. Inclusion criteria included being between the ages of 18–55 years of age and medically cleared for field duty. Exclusion criteria included a current musculoskeletal or neurological condition that would prohibit the completion of performance testing. A 4-week washout period was implemented for anyone who reported current use of supplemental protein and creatine. All participants signed an institutionally approved informed consent form in accordance with the University of Wisconsin La Crosse’s Institutional Review Board (Approved on: 2 June 2023; IRB# 23-KE-137) and Human Subject Research Guidelines.

### 2.3. Procedures

#### 2.3.1. Anthropometrics

During baseline testing, height and weight were recorded using a stadiometer and portable scale, followed by a body composition assessment using a portable multi-frequency bioelectrical impedance analyzer (H2ON, InBody Inc., Cerritos, CA, USA).

#### 2.3.2. Performance Testing

Participants then completed a maximal effort, 3.5 km time trial on an air-braked cycle ergometer (Assault Bike, Assault Fitness Products, Carlsbad, CA, USA). Firefighters were instructed to complete the time trial as fast as possible. Time to completion (s) was recorded. On a separate day (within 48 h of the time trial), firefighters completed a battery of occupation-specific firefighter tasks. These tasks included a hose carry, body drag, stair climb, and forcible entry (Keiser sled hammer) for time. For the hose carry, firefighters advanced a 30.48 m section of a charged 4.45 cm hose line over a distance of 30.5 m in a straight line before flowing water for 2 s. Rescue (body drag) consisted of firefighters being instructed to grasp a mannequin (mass 50 kg, height: 180 cm) underneath the shoulders using a “seatbelt” grip and dragging the mannequin 30.5 m backward. Stair climb consisted of climbing four flights of stairs and returning to the bottom as quickly as possible. In the forcible entry, firefighters struck a simulated forcible entry chopping device (Keiser FORCE Machine, Keiser Co., Fresno, CA, USA) using a 3.6 kg sledgehammer until completed. Thirty seconds of rest was provided between each task to allow for standardized time for set up and preparation. The total time to complete each task was recorded in addition to the total completion time for all tasks summed together. All testing was completed in non-protective gear attire and without a self-contained breathing apparatus.

#### 2.3.3. Dietary Supplementation

Participants were assigned to ingest a single serving daily of either (A) a 25 g dose of whey protein isolate + 25 g dose of carbohydrate powder (ProCarb); or (B) a 25 g dose of whey protein isolate + 25 g dose of carbohydrate powder (+5 g dose of creatine monohydrate) (Creatine) for a ~24-day period (average supplementation duration was 23 ± 2 days; minimum days = 21, maximum days = 26). All supplements were provided to participants in powder form and were of similar texture, bitterness, appearance, and sweetness. All supplements were weighed and blinded by research personnel not involved in testing. The groups were instructed to ingest the supplements daily, within one hour after exercise on training days, and on non-training days first thing in the morning (immediately upon waking). The whey protein isolate and maltodextrin were provided by Argopur Dairy Cooperative (La Crosse, WI, USA) and the creatine was sourced from 1st Phorm, LLC (St. Louis, MO, USA).

## 3. Statistical Analysis

All analyses were completed using the Statistical Package for the Social Sciences (v26; SPSS Inc., Chicago, IL, USA). Primary outcome measures for this investigation were time to completion for the firefighter-specific tasks and time trial. A 2 × 2 mixed factorial (group × time) ANOVA with repeated measures on time was used to determine any statistically significant differences for time and group main effects and group × time interaction effects. All data are presented as means ± standard deviations. In the event of a significant interaction effect, we conducted a follow-up analysis by calculating delta values (“change scores”) between pre-and post-testing and performed an independent samples *t*-test to examine between-group differences. Data are reported using mean differences and 95% confidence intervals where appropriate. Significance was set at *p* < 0.05.

## 4. Results

No significant differences in measures of performance were observed at baseline (*p* > 0.05). There was a significant main effect for time observed for rescue, stair climb, and total time to completion (*p* < 0.05). There was a significant group × time interaction for rescue and forcible entry (Table 1).

**Figure 2:**
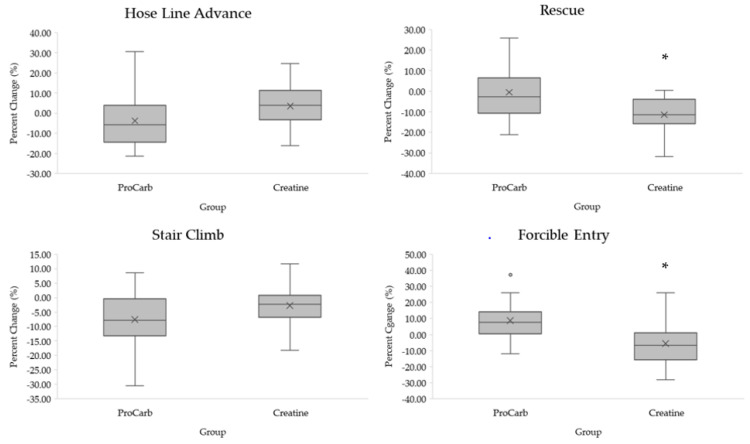
Percent changes in individual occupational performance tasks. * Denotes significance at *p* < 0.05.

Independent sample *t*-tests indicated that the Creatine group recorded a greater reduction in completion time for the rescue (mean difference, 95% confidence intervals; 1.78 ± 0.57 s, 95% CI: 0.61, 2.95 s, *p* = 0.004) and for the forcible entry (2.66 ± 0.97 s, 95% CI: 0.68, 4.65 s, *p* = 0.01) compared to the ProCarb group (Figure 2). No significant group × time interactions were observed for the hose line advance, stair climb, or total time to completion (*p* > 0.05).

**Figure 3:**
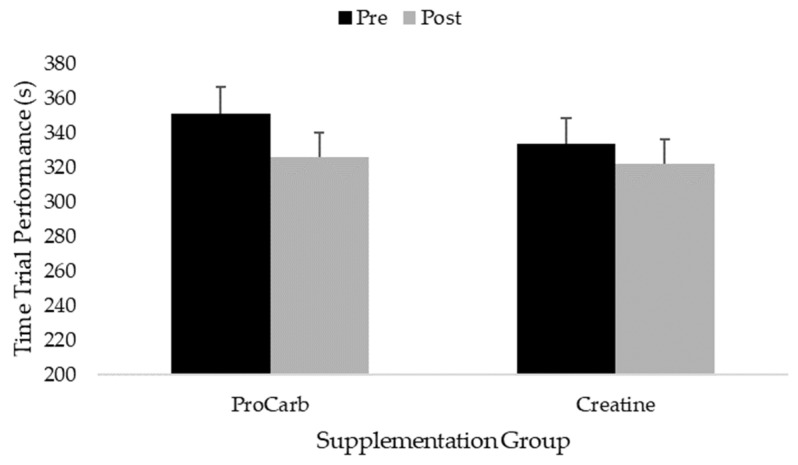
Changes in 3.5 km cycling time trial performance.

There was a main effect for time observed for the time trial (*p* < 0.001), with all firefighters recording faster times during post-testing when collapsed across both groups (Figure 3). No significant group × time interaction was observed (*p* = 0.071). In some cases, mild, acute gastrointestinal distress was reported by participants in the ProCarb (*n* = 1) and Creatine (*n* = 2) groups during the study period. The participants continued the supplementation protocol and did not indicate that it interfered with work-related activities or study procedures.

## 5. Discussion

The primary aim of the current study was to examine the effects of adding creatine to protein and carbohydrate supplementation over a period of 3 weeks on changes in occupational performance in firefighters. The study’s main findings were that providing a daily protein and carbohydrate supplement to the diet over a three week period improved select measures of occupational performance in firefighters. Specifically, improvements in completion times for rescue, stair climb, overall time to completion for all tasks, and time trial performance were observed post testing. Further, the addition of creatine led to greater improvements in time to completion for the rescue and forcible entry tasks, compared to protein and carbohydrate supplementation alone. This study is the first of its kind to report on the ergogenic benefits of providing firefighters with dietary ingredients purported to enhance occupational-specific performance outcomes.

Because of the physical demands of their occupation, it is recommended that tactical populations adhere to specialized dietary recommendations designed to meet their activity levels and fitness-related training goals [8]. These nutritional recommendations often include greater energy and macronutrient intake, along with the implementation of nutrient timing strategies specific to protein and carbohydrates, with an emphasis on the peri-workout window. Even supplementation with whey protein alone has been shown to confer favorable improvements on various measures of performance in trained individuals [35]. In the current study, the daily provision of supplemental protein and carbohydrates led to favorable improvements in key performance tasks, which may have been mediated by improvements in fuel availability throughout each day, along with adequate amino acids to support protein synthesis and overall recovery throughout their weekly training activities. As such, this could have underpinned the mechanisms behind the improvements in select measures of occupational performance (Table 1) observed in the current study. Previous work in tactical populations has reported mixed results regarding similar nutritional interventions. Walker et al. [36] examined the effects of whey protein supplementation over a period of 8 weeks on physical performance for U.S. Air Force personnel. The participants were engaged in their normal training activities (3 days·week^−1^ of aerobic and muscle endurance training) throughout the study period. Improvements in select measures of performance were observed during post-testing; however, no differences were observed between groups. A limitation of the study is that the training programs were not standardized, which may have influenced the acute training stimuli and overall adaptive response throughout the study period. In contrast, a study by McAdam et al. [37] reported greater improvements in push-up performance during a two-minute maximal push-up test among Army personnel supplementing with whey protein during Army Initial Entry Training compared to those supplementing with carbohydrates alone (~7 repetitions higher in the WP compared to the CHO group, *p* < 0.001). However, no differences were observed between groups for run-time performance during a two-minute time trial. It is also worth noting that improvements in strength measures have been observed independent of protein and creatine supplementation in men engaged in a resistance training program [32], suggesting that an appropriately designed, periodized training program may be the top priority for eliciting positive training adaptations, regardless of supplementation strategies. More work is needed to examine the effects of long-term dietary interventions on measures of performance in tactical populations.

The addition of creatine was found to further improve select measures of performance for the firefighters included in the current study. Several of the occupational tasks completed in the current study can be characterized as short-duration bouts of maximal effort. Specifically, average completion times for the tasks completed in the current study were 9–30 s in duration, with the exception of the time trial which lasted ~340 s in duration. We previously reported that this type of firefighter circuit elicits mean heart rate values of 86.8 ± 6.3% of age-predicted max heart rates, with peak values reaching 98.9 ± 5.6% of maximum heart rate [4]. As such, these tasks likely place a high demand on the phosphocreatine and anaerobic energy systems [38]. Further, each of the tasks selected is commonly used during firefighting training and simulations as they are designed to closely mimic on-the-job performance tasks. Thereby, sound rationale exists for the potential ergogenic benefit of creatine for this population. In the current study, the addition of creatine led to significant improvements in the ‘rescue’ and ‘forcible entry’ tasks. The average time to completion for these tasks was ~13 s and ~17 s, respectively. Both of which are examples of activities, specifically the duration and intensity of activity, that often benefit from creatine supplementation due to their anaerobic demands, as previous research has reported improvements in performance measures of similar maximal-effort intensity and duration in active individuals following creatine supplementation strategies [20,21]. For example, consistent improvements in high-intensity exercise performance have been reported following creatine supplementation (5–20 g per day, for ~5 days–4 weeks in duration) [13,20]. However, the effects of creatine supplementation on occupation-specific tasks in tactical athlete populations are less clear. Bennett et al. [9] found significant improvements in the number of pull-ups completed in male military personnel following 6 weeks of creatine supplementation (20 g·day^−1^ for 6 days; 6 g·day^−1^ for 4 weeks). In contrast, Armentano et al. [13] failed to observe improvements in push-up performance in U.S. Army men and women volunteers following 7 days of creatine supplementation (20 g·day^−1^). Samadi et al. [12] reported improvements in vertical jump performance when creatine was added during the last week of a 4-week beta-alanine supplementation protocol in military personnel; however, no improvements in a repeated anaerobic sprint test, or power or performance during a casualty evacuation test were seen. To date, this is the first study to examine the effects of creatine supplementation on occupation-specific performance tasks in firefighters. Results from the current study are in alignment with previous findings in tactical populations and indicate that ~24 days of creatine supplementation (5 g·day^−1^) appears to further improve performance in two distinct tasks commonly completed amongst firefighters (rescue and forcible entry tasks); however, performance in other occupational tasks was unaffected by the addition of creatine to the supplementation protocol. A previous study with firefighters reported that the rescue (34.1%) and forcible entry tasks (21.8%) are responsible for the greatest percentage of total intra-task times during a simulation [28]. Therefore, if the time to completion for these tasks can be reduced via creatine supplementation, as was demonstrated in the current study, it will have the greatest reduction in time to complete the circuit. In a profession where every second counts, this could have important implications during fire suppression and rescue activities.

It is unknown why improvements in some performance parameters have been observed while others have reported null findings within the same population across the literature, including what was found in the current study. Variations in training age, physical fitness status, and familiarity with performance tasks may influence outcomes following creatine supplementation. Furthermore, despite the term tactical population being used to characterize military personnel, firefighters, police, and first responders, the heterogeneity of the individuals across these sub-groups, along with the differences in job activities or daily physical activity levels, may preclude the generalizability of findings across all tactical populations. Therefore, it is important for future research to isolate each sub-group to examine the efficacy of various dietary interventions on performance outcomes that are specific to that population and, subsequently, the occupational demands. Even within the fire service, there are well-known differences in the occupational demands of wildland versus structural firefighting because of the contrast in how fire suppression is handled, along with the environmental demands.

Importantly, with the exception of an increase in body mass, which can commonly be attributed to increased fluid retention and increases in fat-free mass, none of the studies reported adverse effects, which is an important indication of the safety of creatine supplementation for this population. In the current study, two participants from the creatine group and one from the ProCarb group reported mild gastrointestinal distress during the supplementation period that resolved on its own, even with continuation of the assigned supplement. It is possible the artificial flavoring agents used in the manufacturing of the supplement caused mild irritation; however, it is difficult to definitively identify the primary causality. This study is not without limitations. For example, this study did not include a true control group in the current design. Additionally, due to the field-based nature of this study, there were no intramuscular measurements of metabolites, so changes in phosphocreatine and total creatine content were not assessed. Lastly, due to the varying schedules of the firefighters, a standardized diet and training program were not imposed throughout the duration of this study. Thus, future research involving firefighters should consider strategies to minimize the potentially confounding factors.

## 6. Conclusions

The addition of supplemental protein and carbohydrates to the diet of career firefighters throughout a three-week period improves occupational performance in specific areas of high-intensity activities. Furthermore, the addition of creatine within the protein and carbohydrate supplementation leads to greater improvements in specific tests when compared to protein and carbohydrates alone. These findings provide preliminary evidence supporting the benefits of targeted dietary strategies for occupational performance benefits in firefighters.

## Figures and Tables

**Table 1:** Summary of time to completion for measures of occupational performance across each group.

Task	Group	Pre	Post	*p* Value
Hose Line Advance (s)	ProCarb (*n* = 14)	9.3 ± 1.3	9.3 ± 2.2	T: 0.333
	Creatine (*n* = 14)	9.5 ± 1.0	10.1 ± 1.6	G × T: 0.353
Rescue (body drag) (s)	ProCarb (*n* = 14)	12.5 ± 4.0	12.5 ± 4.7	T: 0.006 *
	Creatine (*n* = 14)	14.7 ± 2.5	12.9 ± 2.4	G × T: 0.004 *
Stair Climb (s)	ProCarb (*n* = 14)	30.9 ± 6.7	28.2 ± 5.6	T: 0.044 *
	Creatine (*n* = 14)	30.3 ± 5.0	30.1 ± 3.6	G × T: 0.064
Forcible Entry (s)	ProCarb (*n* = 14)	15.8 ± 4.4	16.7 ± 5.1	T: 0.960
	Creatine (*n* = 14)	18.1 ± 6.3	17.7 ± 6.1	G × T: 0.010 *
Total (s)	ProCarb (*n* = 14)	68.4 ± 12.0	67.2 ± 14.1	T: 0.042 *
	Creatine (*n* = 14)	72.6 ± 12.4	69.9 ± 10.8	G × T: 0.452

* Denotes significance at *p* < 0.05. Data presented as mean ± SD; T = time effect; G × T = group × time interaction.

## Data Availability

Data are available upon request.
